# Role of Social Media in Diabetes Management in the Middle East Region: Systematic Review

**DOI:** 10.2196/jmir.9190

**Published:** 2018-02-13

**Authors:** Turki Alanzi

**Affiliations:** ^1^ Department of Health Information Management and Technology College of Public Health Imam Abdulrahman Bin Faisal University Dammam Saudi Arabia

**Keywords:** social media, Saudi Arabia, eHealth, telemedicine, mobile phone, cell phone, outcome of care, Middle East

## Abstract

**Background:**

Diabetes is a major health care burden in the Middle East region. Social networking tools can contribute to the management of diabetes with improved educational and care outcomes using these popular tools in the region.

**Objective:**

The objective of this review was to evaluate the impact of social networking interventions on the improvement of diabetes management and health outcomes in patients with diabetes in the Middle East.

**Methods:**

Peer-reviewed articles from PubMed (1990-2017) and Google Scholar (1990-2017) were identified using various combinations of predefined terms and search criteria. The main inclusion criterion consisted of the use of social networking apps on mobile phones as the primary intervention. Outcomes were grouped according to study design, type of diabetes, category of technological intervention, location, and sample size.

**Results:**

This review included 5 articles evaluating the use of social media tools in the management of diabetes in the Middle East. In most studies, the acceptance rate for the use of social networking to optimize the management of diabetes was relatively high. Diabetes-specific management tools such as the Saudi Arabia Networking for Aiding Diabetes and Diabetes Intelligent Management System for Iraq systems helped collect patient information and lower hemoglobin A_1c_ (HbA_1c_) levels, respectively.

**Conclusions:**

The reviewed studies demonstrated the potential of social networking tools being adopted in regions in the Middle East to improve the management of diabetes. Future studies consisting of larger sample sizes spanning multiple regions would provide further insight into the use of social media for improving patient outcomes.

## Introduction

Diabetes mellitus is a group of metabolic disorders characterized by high blood glucose levels and inflammation leading to long-term complications of obesity, heart disease, stroke, foot ulcers, and loss of eyesight and kidney function [1,2]. After a person consumes a meal, the pancreas releases the hormone insulin, which binds to cell receptors to signal uptake of glucose from the blood. However, in diabetes, the cells are unable to take up glucose from the blood. Type 1 and type 2 are the 2 most common forms of diabetes mellitus. Type 1 diabetes occurs due to the inability of the pancreas to produce insulin, whereas type 2 diabetes occurs due to cells developing a resistance to insulin.

Diabetes prevalence is rapidly increasing in developing countries, including those in South Asia, the Middle East, Sub–Saharan Africa, and Latin America [3]. Diabetes has various risk factors, including obesity for type 2 diabetes, where the body progressively develops insulin resistance [4]. In addition, there is an ethnic predisposition to type 2 diabetes that is most notably present in Asians [5]. A special case of concern is for the population in the Middle East, with one of the highest prevalences of overweight [5] due to a high prevalence of physical inactivity [6]. The countries in this region have boasted rapid economic growth in the last three decades. This has led to changes in lifestyle, with lower physical activity levels and an increase in dietary caloric intake, resulting in high incidences of obesity, diabetes, and cardiovascular diseases. For example, one nationwide study in Iran showed that the occurrence of metabolic syndrome is upward of 35%, and is higher in women and populations in urban areas [5]. This pattern is expected to impose an enormous burden on the health care system in the Middle East region and calls for an implementation of improved methods of self-care for disease management. Several large-scale clinical randomized trials have shown that type 2 diabetes can be prevented or the effects can be minimized through self-care by changing one’s lifestyle with a low-calorie diet and increased physical activity [7].

Due to recent technological advances, more and more people are relying on new communication channels, such as online information and social media, than on traditional messages on television and in print media [8]. Consequently, there has been a growing effort to introduce efficient means of implementing electronic and digital processes in health care. This correlates with the high use of smartphones and the popularity of mobile phone–based apps developed for virtually all aspects of life. Social networking is dominated by apps, such as Facebook, Twitter, Snapchat, Instagram, and YouTube, with millions of subscribers. These apps have provided a means of communicating quickly and efficiently with the masses, at minimal cost, in a very short period of time. In health care, social networking is garnering more users, and its benefits in educating patients about their disease, dietary limitations, and physical activity are already proving their worth [8-11]. Due to high economic growth and the presence of a reliable network in the Middle East region, a large proportion of the population in the Middle East region own a smartphone. One example is that the Kingdom of Saudi Arabia (KSA) has the largest percentage of mobile phone users in the world, according to a United Nations Conference on Trade and Development report [12]. The increase in mobile phone use is accompanied by a spread of social networking apps, especially among the young and the educated population of the KSA.

Due to the far-reaching presence and ease of use of mobile phones and social media, many researchers and health care providers are encouraging the use of mobile phones in the Middle East to better educate patients on how to manage their disease. Previously, several studies had shown the benefit of using social media campaigns to educate patients about diabetes, obesity, antibiotic use, and adolescent dating violence [9,13,14]. For health promotion advocates, social media presents a platform that is highly cost efficient and delivers mass education that ultimately is responsible for lowering the burden on regional health care systems. From the user’s perspective, social media platforms have several benefits, such as making open access information available, providing the option for dynamic conversations in a group, and keeping users connected with their topic of interest [13]. This paper presents the evidence on evaluating the impact of social media intervention in improving the health of patients with diabetes in the Middle East.

## Methods

### Search Strategy

Both PubMed (1990-2017) and Google Scholar (1990-2017) were searched for research studies using various combinations of the following search terms: “diabetes,” “diabetes mellitus,” “mobile phone,” “cell phone,” “cellular phone,” “social media,” “social network,” “Facebook,” “Twitter,” and “Snapchat.” References of the identified articles were also searched for potential articles for inclusion. Only articles published in English in peer-reviewed scientific journals were eligible for review. The main inclusion criterion was that studies used mobile social networking apps for diabetes care in the Middle East as the primary intervention approach.

### Study Selection

The search successfully identified 26 unique articles that addressed the basic criterion of the search: diabetes with the use of mobile phones in the Middle East. A review of the titles and abstracts of all relevant search results identified 15 articles that were relevant to the objectives of this review and met the following inclusion criteria: (1) randomized controlled trials, (2) quasi-experimental studies, and (3) pre-post studies evaluating the use of mobile phones for improving the education and health care of patients with diabetes in the Middle East. Further refining of the search results excluded articles that did not incorporate a social network app in their study (n=9) or did not specify a location (n=1). A final selection of 5 articles [12,15-18] that met the above-mentioned criteria were included for the review and analysis. A thorough review of all articles collected the following information: descriptions of the study design, country in which the study was conducted, sample size, patient age, duration of the study, technology used, frequency of intervention, method of intervention, control groups, self-care management activities, educational content and delivery, process and outcome measures, and statistical significance.

## Results

### Study Designs and Participants

The participants in the 5 studies included in this review were primarily patients with type 2 diabetes (80% of the studies), while the other patients had both type 1 and type 2 diabetes. The duration of diabetes was reported in 3 of the selected studies [12,17,18], whereas additional clinical data such as hemoglobin A_1c_ (HbA_1c_), body weight, body mass index, and cholesterol levels were reported in only 1 study [18]. In most of the studies, the sample sizes ranged from 12 to 33 participants, with ages extending from 10 to 68 years. However, in 1 particular study, the participant sample size was not directly assessed, as the method of data collection consisted of compiling information from 7 groups for a total of 1551 Facebook posts [17]. In this study, participant ages ranged from less than 20 to 80 years.

Most of the reviewed studies used mixed methods of design interviews and surveys to gather data regarding the acceptance of social media for the management of diabetes. In 1 study [17], a mixed-methods quantitative and qualitative content analysis was conducted to assess data collected from Facebook posts. In other studies [12,15], the Questionnaire for User Interaction Satisfaction assessed users’ satisfaction according to a 9-point Likert scale after using the Saudi Arabia Networking for Aiding Diabetes (SANAD) system [15].

Furthermore, in 1 study, patients randomly allocated to 2 groups (intervention vs control) were selected to evaluate the effectiveness of the Diabetes Intelligent Management System for Iraq (DIAR) system for mobile diabetes management [18]. Most of the selected studies were conducted in the KSA. However, 1 study [18] was conducted in Iraq, whereas another study [17] included data from a series of 22 Arabic-speaking countries. Multimedia Appendix 1 lists the reviewed studies and the social networking intervention used, together with each of the Middle Eastern countries where these studies were conducted.

### Social Networking Apps in the Middle East

Social networking apps used to facilitate the management of diabetes from the patient’s perspective included the SANAD system [12,15]. The SANAD system has 3 main entities: (1) a mobile diabetes management module, (2) a social networking module, and (3) a cognitive behavioral therapy module for behavioral change issues. Another study used the DIAR system for mobile diabetes management [3]. The DIAR system consisted of 2 main components: (1) a mobile self-monitoring of blood glucose system and (2) a remote Web interface and health management system. Other social network tools consisted of mobile phones [16] and social networking media such as Facebook [17].

### Social Networking System in the Kingdom of Saudi Arabia

This review focused on the benefits of social networking and mobile diabetes management in the patient population of the Middle East region. In the first example, my group presented a mobile social networking system, SANAD, tailored for patients with type 2 diabetes in the KSA [12,15]. SANAD was designed to provide a smart social behavioral change intervention and management for Saudi patients with diabetes. To evaluate this system, 33 patients with diabetes were surveyed, and 80% indicated that the SANAD mobile system was effective in managing their diabetes [12,15]. However, the patients indicated that aspects relating to terminology and system information needed improvement.

In another study, we showed that a large number of patients in the KSA were open to using social networking as a tool for better management of their diabetes [16]. Specifically, the patients preferred the features that allowed them to speak to and obtain feedback from a medical professional in real time.

### mHealth System in Iraq

To assess the effectiveness of a mobile health study in a conflict region, Istepanian et al designed and implemented a feasibility study on mobile diabetes management in Basra, southern Iraq [18]. This pilot study was set up as a model to test the effectiveness of mobile health technologies to improve health care in other postconflict regions. The study recruited a total of 12 patients with type 2 diabetes from 1 hospital. The patients were evaluated for HbA_1c_, low-density lipoprotein cholesterol, and high-density lipoprotein cholesterol before and after commencing the study. The results showed a significant decrease in HbA_1c_ levels; however, no change was observed in other parameters. Overall, the patients were satisfied with the mobile health intervention and wished to continue it as part of their medical care [18]. The major limitation of this study was the small sample size and lack of regional diversity.

### Social Media Platforms in Arabic-Speaking Countries

A study by AlQarni et al assessed the sharing of diabetes-related health information on the social media platform Facebook [17]. This study analyzed a total of 1551 Facebook posts originating in 22 countries. The principal focus of the posts was on sharing personal experiences with diabetes (n=423, 27.3%), followed by posts supporting patients and caregivers (n=220, 14.2%), raising awareness of the condition (n=210, 3.5%), providing spiritual support (n=162, 10.4%), sharing the latest research (n=147, 9.5%), and providing education (n=110, 7.1%) on diabetes. A large proportion of the posts by individuals aged between 40 and 60 years focused on finding out diagnosis-related information due to limited access to care in their home countries. These findings support the increasing rate of sharing information on social media to improve public health.

## Discussion

### Principal Findings

The purpose of this review was to assess the impact of social network platforms on the control and management of diabetes in the Middle East region. The use of technological devices as a means of providing effective health care solutions is rapidly increasing, especially in low- to middle-income countries [18]. Health education and awareness programs are being recognized as key players responsible for this rising trend. The studies evaluated in this review demonstrated that the acceptance of social networking tools for better management of diabetes is relatively high in the Middle East. Generally, newer networking system such as SANAD and DIAR, tailored for individuals with diabetes, were easily adopted and received high ratings. More specifically, patients reported a favorable overall impression and satisfaction in terms of aspects of screening and learning, and capabilities of the system. However, patients rated aspects of terminology and system information and learning factors poorly. Improving the factors that caused user dissatisfaction would promote further use of social networking tools to support disease management.

As in the case of more universal social media avenues such as Facebook, it is evident that the sharing of health information, especially for chronic diseases with a high global prevalence, such as diabetes, is also increasing. Patients are finding new ways to find answers via social media platforms to address health concerns with other users. It is noteworthy that there are several advantages to using social networking for the management of a disease. However, without the proper regulations and screening tools for social media sites in place, there is the potential of acquiring misleading management advice from an erroneous source.

From a clinical standpoint, provision of improved glycemic control and education through mobile phone technologies is an effective and available approach to provide knowledge to patients with type 2 diabetes. However, limited resources and deployment options are some of the challenges to getting the devices to areas in the Middle East region.

This review highlights that social media networking interventions are considered as acceptable approaches to improving management of and education about diabetes. However, there are some limitations that should be noted. Some of the studies included in the assessment did not report power calculations. Furthermore, several studies assessed in this review contained small sample sizes limited to 1 region. Therefore, further studies with larger sample sizes and spanning multiple cities and regions are required to fully evaluate the social network interventions for the management of diabetes.

### Conclusion

This review evaluated the available data and studies of the impact of social networking on diabetes management in Middle Eastern countries. Considering the importance and the massive popularity of social networking tools in the region, only a few studies to date have addressed this important health care area. However, these studies indicated the potential impact of social networking tools in improving diabetes care and outcomes. Further studies consisting of larger sample sizes spanning multiple regions would provide further insight into the use of social network media for improving outcomes of patients with diabetes.

## Appendix

Multimedia Appendix 1 List of reviewed studies.

## Abbreviations

DIAR: Diabetes Intelligent Management System for Iraq
HbA_1c_: hemoglobin A_1c_
KSA: Kingdom of Saudi Arabia
SANAD: Saudi Arabia Networking for Aiding Diabetes
