# Supplementary material for: Role of Social Media in Diabetes Management in the Middle East Region: Systematic Review
Source: J Med Internet Res. 2018 Feb 13;20(2):e58. doi: 10.2196/jmir.9190 (PMC5829453; doi:10.2196/jmir.9190)
Supplement: Multimedia Appendix 1 [file jmir_v20i2e58_app1.pdf]

Table 1. The list of the reviewed studies and the type of the social networking interventions in the ME countries

Author/ year	Study type	Number of participants	Type of Diabetes	Study location	Type of technological intervention	Results summary
Alanzi, 2014	Mixed methods	30	1 and 2	Saudi Arabia and United Kingdom	Social networking using cell phone	Relatively high number of Saudi patients accept using social networking as a tool for better managements of their diabetes.
Alanzi, 2014	Quasi-experimental Pre-post	33	2	Saudi Arabia	Mobile app: Saudi Arabia Networking Aiding Diabetes (SANAD) system	The SANAD system used a smartphone diabetes management module to collect blood glucose records from diabetes patients. A higher satisfaction with the overall impression, and satisfaction including screen, learning, and capabilities aspects of the system. However, poor ratings were obtained in aspects relating to terminology and system information and learning factors.
Istepanian, 2014	Quasi-experimental Pre-post	12	2	Iraq	DIAR mobile diabetes self-management system	The DIAR system lowered the HbA1c level in the intervention group from 8.95% to 8.05%. The outcome of the questionnaire survey showed that all patients were satisfied with mobile health intervention and agreed that the service should be continued as part of their medical care after the study.
Alanzi, 2016	Mixed methods	33	2	Saudi Arabia	Mobile app: Saudi Arabia Networking	Higher satisfaction with the overall impression, satisfaction, being stimulated, ease of use, perceived

					Aiding Diabetes (SANAD) system	powerfulness, and flexibility.
AlQarni, 2016	Exploratory study	1551 Facebook posts	1 & 2	Arab world	Facebook	The focus of the posts was on sharing personal experiences (27%), raising awareness (3.5%), providing spiritual support (10.4%), sharing latest research (9.5%), and providing education (7.1%).
